# Mesoporous Core–Cone Silica Nanoparticles Can Deliver miRNA-26a to Macrophages to Exert Immunomodulatory Effects on Osteogenesis In Vitro

**DOI:** 10.3390/nano13111755

**Published:** 2023-05-29

**Authors:** Sepanta Hosseinpour, Huan Dai, Laurence J. Walsh, Chun Xu

**Affiliations:** School of Dentistry, The University of Queensland, Herston, QLD 4006, Australia

**Keywords:** mesoporous silica nanoparticles, microRNA-26a, immunomodulation, osteogenesis, macrophage

## Abstract

Nanoparticles can play valuable roles in delivering nucleic acids, including microRNAs (miRNA), which are small, non-coding RNA segments. In this way, nanoparticles may exert post-transcriptional regulatory influences on various inflammatory conditions and bone disorders. This study used biocompatible, core–cone-structured, mesoporous silica nanoparticles (MSN-CC) to deliver miRNA-26a to macrophages in order to influence osteogenesis in vitro. The loaded nanoparticles (MSN-CC-miRNA-26) showed low-level toxicity towards macrophages (RAW 264.7 cells) and were internalized efficiently, causing the reduced expression of pro-inflammatory cytokines, as seen via real-time PCR and cytokine immunoassays. The conditioned macrophages created a favorable osteoimmune environment for MC3T3-E1 preosteoblasts, driving osteogenic differentiation with enhanced osteogenic marker expression, alkaline phosphatase (ALP) production, extracellular matrix formation, and calcium deposition. An indirect co-culture system revealed that direct osteogenic induction and immunomodulation by MSN-CC-miRNA-26a synergistically increased bone production due to the crosstalk between MSN-CC-miRNA-26a-conditioned macrophages and MSN-CC-miRNA-26a-treated preosteoblasts. These findings demonstrate the value of nanoparticle delivery of miR-NA-26a using MSN-CC for suppressing the production of pro-inflammatory cytokines with macrophages and for driving osteogenic differentiation in preosteoblasts via osteoimmune modulation.

## 1. Introduction

Nanoparticles can have a range of applications, and amongst these, there may be great value in using them to alter inflammatory processes and to promote healing. Inflammation is a double-edged sword for bone healing. The initial optimal transient stage of acute inflammation is essential for the sufficient regeneration of bone to occur [1]. On the other hand, uncontrolled inflammation can cause tissue damage and inhibit healing. Excessively long or severe acute inflammation can be caused by infections, surgical interventions, or thermal, chemical, or mechanical injuries [2]. This then drives the excessive secretion of pro-inflammatory cytokines [2,3], especially from macrophages. Cytokines such as tumor necrosis factor (TNF) and interleukin (IL)-6–α inhibit mesenchymal stem cell differentiation [4], stimulate osteoclastogenesis, and increase nuclear factor kappa B (NF-κB) pathway activity [5].

For optimal bone regeneration, one must consider how an applied biomaterial could suppress deleterious host immune responses, whilst at the same time, modulating the immune response to promote healing. This means a design concept shift from inert biomaterials to those capable of producing immunomodulatory effects [6,7,8]. Hence, modern nanomaterial concepts should apply an “osteoimmunomodulation” knowledge framework [9]. Successful bone regeneration requires the biomaterial to interact with immune cells, avoiding ‘immunotoxicity’, and rather, allowing it to be used for ‘immunotherapy’ [10,11]. In this context, we designed a delivery system based on a novel nanovector, comprising core–cone mesoporous silica nanoparticles (MSN-CC). These nanoparticles were used to deliver microRNA (miRNA)-26a into macrophages and osteoprogenitor cells, with the overall goal being to enhance bone formation. Once it is released inside the transfected cells, miRNA regulates gene expression at the transcriptional and post-transcriptional levels [12]. For the macrophages, this results in activation and polarization away from an inflammatory phenotype [13,14].

The choice of miRNA cargo for the nanoparticle vector in our study was based on evidence for a prominent role of miRNAs in influencing macrophage activation and differentiation (reviewed in [15]). MiRNA-26a is known to reduce the expression levels of IL-1β, IL-6, and TNF-α in microglial cells exposed to lipopolysaccharide (LPS) [16]. It also influences the cyclooxygenase-2 pathway and reduces the synthesis of prostaglandin E2 [17]. In terms of the effects on bone cells, miRNA-26a modulates bone formation via its effects on the Wnt and BMP/Smad signaling pathways [18,19]. There is, at present, no direct evidence for osteoimmunomodulation from this miRNA; hence, we wished to explore its immunomodulatory impacts on osteogenesis.

When one is considering a suitable carrier for miRNA cargo, the key considerations are that the vector must ensure the stability of miRNA in the extracellular matrix/fluid and be taken up into the cytoplasm of the targeted cells. MSN-CC have been used for nucleic acid delivery [20], with a high transfection efficiency and low toxicity. These nanoparticles can undergo a range of surface modifications [20]. MSN can exert immunomodulatory actions themselves on osteoprogenitor cells that are useful in terms of the goal of bone regeneration (reviewed in [20]). The MSN-CC delivery platform has previously been optimized for miRNA delivery into osteoprogenitor cells by our team [19]. In the present study, we assessed the immunomodulatory impact of miRNA-26a delivered by MSN-CC into macrophages and the resulting impacts of this on preosteoblasts in terms of bone production.

## 2. Materials and Methods

### 2.1. Preparation and Surface Modification of the Nanoparticles

MSN-CC were synthesized using a previously reported method [21]. Briefly, 24 mL of 25% aqueous solution of cetyltrimethylammonium chloride (Sigma-Aldrich, St. Louis, MO, USA), 0.2 g of triethanolamine, and 36 mL of Milli-Q water were mixed; then, 17.5 mL of chlorobenzene and 2.5 mL of tetraethyl orthosilicate were added, and the mixture stirred at 500 rpm at a temperature of 60 °C for 12 h. Products were separated via centrifugation and calcined at 550 °C for 5 h. Following the dispersion of 60 mg of nanoparticles in 20 mL of water, a 10 mL volume of 56 mM 3-(trihydroxysilyl) propyl-methyl phosphonate (THPMP) solution was added to the mixture and stirred at 40 °C for 2 h for surface phosphonate modification. Products were collected via centrifugation and resuspended in a polyethyleneimine (PEI) solution, which was prepared via mixing 150 mg of PEI (10 kD) with 15 mL of 100 mM carbonate buffer (pH 9.6). The suspension was stirred at room temperature for 4 h. Finally, after centrifugation, PEI-coated nanoparticles were washed and dried at room temperature.

### 2.2. Characterization of Nanoparticles

The size and morphology of MSN-CC after PEI coating were observed with a transmission electron microscopy (TEM) (model HT7700, Hitachi, Tokyo, Japan). 

### 2.3. Preparation of Nanoparticle-microRNA Complexes

MSN-CC-PEI (10, 20, or 40 μg) were incubated in 50 μL of media without serum with 30 pmol of carboxyfluorescein (6-FAM), which was labelled miRNA (miRNA-26a-5p mimic/inhibitory) or plain negative control (NC) miRNA, for 15 min. All miRNA samples were purchased from GenePharma (Shanghai, China). The negative control miRNA used was a non-functional sequence of 23 nucleotides.

### 2.4. Cell Culture

A murine-derived macrophage cell line (RAW 264.7 cells) and a preosteoblastic murine-derived cell line (MC3T3-E1, derived from mouse C57BL/6 calvaria, 99072810) were purchased from the American Type Culture Collection (ATCC) and from CellBank Australia (Westmead, NSW, Australia), respectively. Both cell types were cultured in high glucose Dulbecco’s modified Eagle’s medium (DMEM) (Life Technologies, Carlsbad, CA, USA) supplemented with 10% fetal bovine serum (Thermofisher Scientific Australia, Scoresby, VIC, Australia) and 1% penicillin and streptomycin (Gibco), and then incubated at 37 °C in 5% CO_2_. Cells were frequently passaged at approximately 80% confluency and used for three to five passages. 

### 2.5. Immunotoxicity Tests

Macrophages were seeded in 24-well plates at a density of 1 × 10^4^ cells/mL and cultured in DMEM with 15% FBS and 1% penicillin-streptomycin for 24 h, before being exposed to MSN-CC or MSN-CC-PEI at different concentrations (5, 10, 20, 40, 80, 160, 320, and 640 μg/mL). Untreated cells were used as a negative control. After 1, 3, and 7 days, 10 μL 3-(4,5-dimethylthiazol-2-yl)-2, 5-diphenyltetrazolium bromide (MTT) (Sigma, St. Louis, MO, USA) (5 mg/mL) was added to each well, and the cells were incubated at 37 °C in 5% CO_2_ for another 4 h. After incubation, the reaction was terminated via adding dimethyl sulfoxide (DMSO) (Roche, Basel, Switzerland), and the plates were shaken for 15 min in the dark. Optical absorbance was read with a microplate reader (Infinite, Tecan Trading AG, Männedorf, Switzerland) at a wavelength of 565 nm, and the percentage of viable cells was calculated. In addition, MC3T3 cells and macrophages were cultured under the same conditions as mentioned above and exposed to 10, 20, and 40 μg/mL of it (the same concentrations as used for transfection). The proportions of live and dead cells were evaluated using a Live/Dead double staining kit (LIVE/DEAD™ Viability/Cytotoxicity Kit, Invitrogen™, Thermo Fisher Scientific, Burlington, ONT, Canada). After culturing with or without MSN in a standard medium or in the macrophage-conditioned medium (CM) for 1 and 3 days, the cells were stained in a medium containing calcein-AM and ethidium homodimer-1 for 30 min, which was followed by the acquisition of representative cell images using a confocal laser scanning microscope (CLSM) (Nikon C2+, Nikon, Tokyo, Japan).

### 2.6. Transfection Efficiency and Cellular Uptake

Known amounts (10, 20, and 40 µg/mL) of MSN-CC-PEI-FAM-miRNA were used to transfect macrophages (1 × 10^4^ per mL cell density) for 4 h. Lipofectamine™ 3000 reagent (Life Technologies, Carlsbad, CA, USA) was used as a positive control following the manufacturer’s guidelines. Transfection efficiency and cellular uptake were measured via CLSM and flow cytometry. For CLSM observations, macrophages were fixed for 30 min in 4% paraformaldehyde, after which time, the cells were permeabilized with 0.1% Triton X (J.T. Baker, Phillipsburg, NJ, USA) for 10 min and washed three times in PBS. Nuclei were stained with DAPI (4′,6-diamidino-2-phenylindole) (D1306, Thermo Fisher Scientific), while actin filaments (in the cytoskeleton) were stained with phalloidin (Alexa Fluor^®^ 555, Thermo Fisher Scientific) for 30 min. Lastly, the samples were mounted on glass slides and examined with a confocal microscope (Nikon C2+, Nikon, Tokyo, Japan). The intracellular distribution of FAM-labelled miRNA in each group was revealed via 488 nm laser excitation. For flow cytometry, after transfection, cells were trypsinized and washed with 0.15 M phosphate-buffered saline (PBS). Following the fixation of samples with paraformaldehyde, the number of FAM-positive cells was quantified using a flow cytometer (FACS Canto II, BD Biosciences, San Jose, CA, USA), with 1000 cells per sample, using excitation at 488 nm. FlowJo software, version 10.6.2 (FlowJo LLC, Ashland, OR, USA), was used to analyze flow cytometry data.

### 2.7. Assessment of the Modulatory Effects of Nanocomplexes on Macrophage

Macrophages were seeded in a 6-well plate at a density of 10^6^ cells/well. Cells were transfected with MSN-CC-PEI-miRNA-26a-5p mimic/inhibitory or the MSN-CC-PEI-plain negative control (NC)-miRNA, as described in Section 2.7. 

After incubation for 1 and 3 days, the expression level of Wnt5a/Ca^2+^ pathway-related genes (*Wnt5a* and frizzled class receptor 5 (*Fzd5*)), pro-inflammatory cytokines (IL-1β and IL-6), an anti-inflammatory marker (IL-10), M1 surface markers CD11c and CD86, and the M2 surface marker CD206 were evaluated via quantitative real-time PCR (qRT-PCR). The primers used are listed in Table 1. Briefly, total RNA was isolated with trizol (Invitrogen™, Thermo Fisher Scientific, Australia). The extracted RNA was measured using a NanoDrop spectrophotometer (Thermo Scientific NanoDrop Products, Wilmington, DE, USA). Two ng of total RNA in each sample was used to create cDNA using Superscript II reverse transcriptase (Invitrogen). 

Real-time PCR was conducted using the SYBR Green PCR Master Mix (Applied Biosystems, Warrington, Cheshire, UK). Amplification curves for the reactions were assessed using LightCycler Software^®^, version 3.5 (Roche Molecular Biochemicals). The comparative CT method was used for relative qualification, and relative gene expression (2-ΔΔCT) was determined and used to calculate fold-change differences between the control and differentiated cultures using the Gene Globe Analysis application (http://www.qiagen.com/geneglobe accessed on 10 February 2022). Glyceraldehyde 3-phosphate dehydrogenase (GAPDH) was used as a housekeeping gene, and its mRNA level was used to normalize results for the target genes of interest. All reactions were performed in triplicate. 

Moreover, the supernatant was collected and centrifuged after 1 and 3 days. The concentrations of IL-1β and TNF-α in the supernatants were examined using enzyme-linked immunosorbent assay kits (Abcam, Cambridge, UK) according to the manufacturer’s instructions.

### 2.8. Assessments of the Modulatory Effects on Preosteoblasts

MC3T3-E1 cells and macrophages were cultured separately in 6-well plates at a density of 10^6^ cells per well. After transfecting the macrophages with the MSN-CC-PEI-miRNA-26a-5p mimic, they were incubated for 14 days, and every three days, the supernatant was collected and used as CM for MC3T3-E1 cells. MC3T3-E1 cells were also transfected with the MSN-CC-PEI-miRNA-26a-5p mimic/inhibitory or MSN-CC-PEI-plain NC (day 0) and were incubated with complete media or CM. A group of cells cultured in the osteogenic medium without exposure to nanoparticles was used as a negative control.

For determining the osteogenic promoting function of CM and comparing it with exposure to MSN-CC-PEI or transfection with the MSN-CC-PEI-miRNA-26a-5p mimic, the relative level of expression of four target genes (Runt-related transcription factor 2 (Runx-2), alkaline phosphatase (*ALPL*), collagen type 1 (Col1α1), osteocalcin (OCN), and receptor activator of nuclear factor kappa-Β ligand (RANKL) (Table 1)) were measured via qRT-PCR, as described in Section 2.8.

### 2.9. ALP Activity Assay and Assessment of Extracellular Matrix Formation and Calcification

After MC3T3-E1 cells were exposed to MSN-CC-PEI or transfected with the MSN-CC-PEI-miRNA-26a-5p mimic, they were cultured with or without CM for 14 days. Untreated cells, which were incubated with or without CM, were used as controls. 

For further osteogenic differentiation assessment, the ALP activity of MC3T3-E1 was assessed (ALP kit, Abcam, Cambridge, UK). Light absorbance at 405 nm was measured with a micro-plate reader (Infinite, Tecan Trading AG, Männedorf, Switzerland). In addition, the deposition of extracellular collagen and the formation of mineralized matrix nodules were assessed via Picro Sirius red and Alizarin red staining (Sigma, St. Louis, MO, USA) after 14 and 21 days, respectively. In brief, cells were fixed in paraformaldehyde solution for 15 min at room temperature, and then washed and stained with 5% Alizarin red or 0.1% Sirius red in saturated picric acid (Electron Microscopy Sciences, Hatfield, PA, USA) for 1 h and examined with an inverted microscope.

To quantify the amount of collagen deposition, stained wells were washed with 0.5 M acetic acid to remove the non-specifically bound dye, and then the bound dye was eluted in 0.5 M sodium hydroxide. Absorbance was measured at 540 nm using a spectrophotometer, and the concentration was calculated by interpolation from a standard curve constructed using known concentrations of dyes. The amount of mineral formed was measured by dilution in acetic acid, as described previously [22], and absorbance was measured at 405 nm using a spectrophotometer. 

### 2.10. Statistical Analysis

All statistical computations were performed using Prism (Version 9.0.0, GraphPad, La Jolla, CA, USA). Data for cell viability (in percentages), immunoassays, gene expression, ALP activity, and quantities of the extracellular matrix were subjected to one-way or two-way analysis of variance (ANOVA) with post hoc Tukey’s tests. A *p* value of less than 0.05 was considered to be statistically significant. Data sets were assessed for normality before parametric statistical analyses were conducted.

## 3. Results

### 3.1. Characterization of MSN-CC with PEI Coating

**Figure 1:**
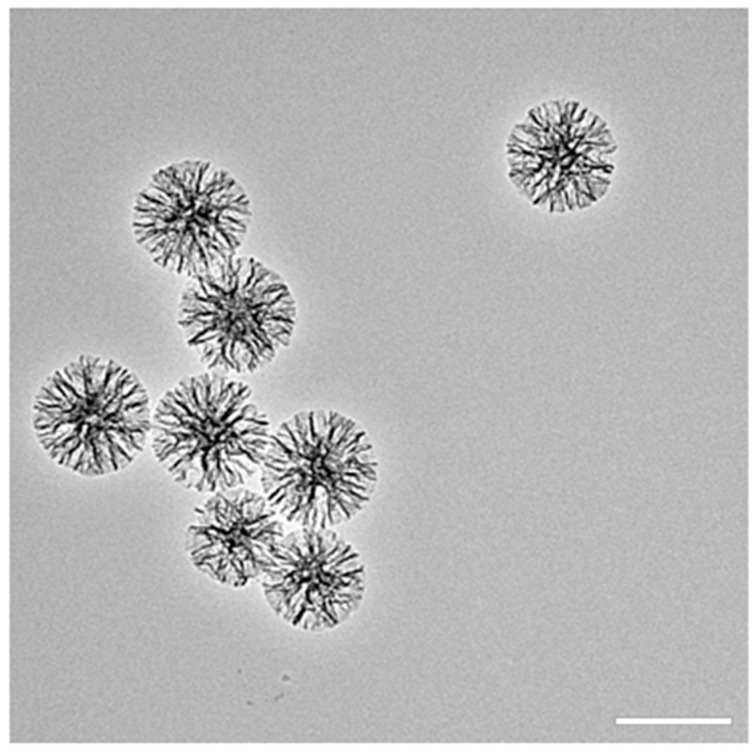
Characterization of MSN-CC-PEI via TEM (scale bar: 250 nm).

The average diameter of MSNs after coating was approximately 200 nm (Figure 1). A well-aligned mesoporous structure was seen via TEM. The morphology, particle size, and pore size were similar between the samples, which appeared to be identical to those of chlorobenzene-water system synthesized particles, which we previously described [21]. 

### 3.2. Cell Viability and Transfection Efficiency of Nanoparticles

**Figure 2:**
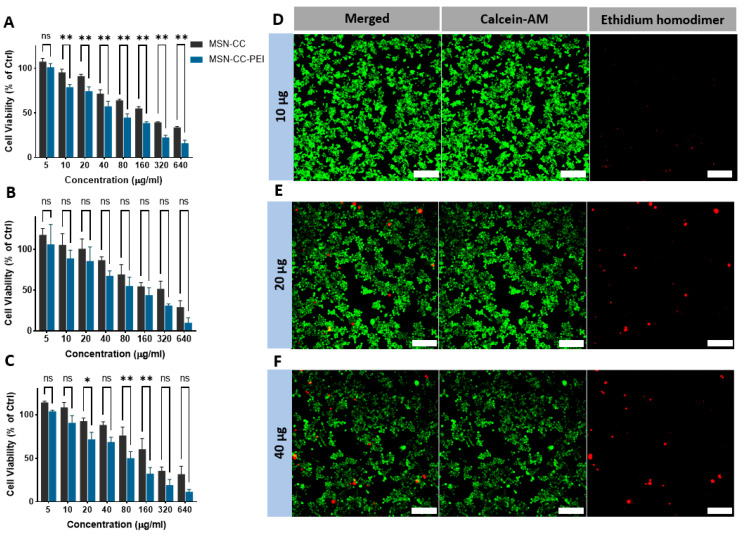
Immunotoxicity of RAW264.7 macrophages after exposure to MSN-CC before and after coating with PEI. MTT assay data after exposure to MSN-CC and MSN-CC-PEI are shown for 1 day (panel **A**), 3 days (panel **B**), and 7 days (panel **C**). ns = no significant difference. * *p* < 0.05; ** *p* < 0.01 via paired *t*-test (*n* = 3). Panels (**D**–**F**) show live and dead staining of RAW264.7 cells exposed to therapeutic doses using ethidium homodimer-1 and the esterase substrate calcein AM (LIVE/DEAD^®^ Cytotoxicity Kit). Live cells are bright green, whereas dead cells with compromised membranes are red. Both assays revealed dose-deponent toxicity, but excellent biocompatibility for concentrations below 40 µg/mL. Scale bars = 100 µm.

MTT and Live/Dead assays demonstrated that MSN-induced cytotoxicity increased in dose- and time-dependent manners (Figure 2 panels A to D). The lowest concentration (5 μg/mL) of MSNs appeared to further increase the absorbance of MTT compared with that of untreated cells (the control), which may be related to MSN, causing enhanced MTT formazan exocytosis [23]. Compared to MSN-CC, MSN-CC-PEI caused more cytotoxicity at doses higher than 40 μg/mL over 1, 3, and 7 days. On days 1, 2, and 3, the percentages of cell viability were lower than 50% for concentrations greater than 80 μg/mL; however, the chosen therapeutic concentrations (<40 μg/mL) caused significantly lower toxic effects on the macrophages (Figure 2A–C). These findings were consistent with the CLSM analysis of Live/Dead cells, as shown in Figure 2 from panels D to F. Although surface functionalization with PEI, as predicted, reduced the percentage of viable cells in a dose-dependently manner, when the PEI-coated nanoparticles were used at a low concentration (i.e., less than 20 µg/mL), the cell viability was high (71.95% ± 6.5 after 3 days, Figure 2C).

**Figure 3:**
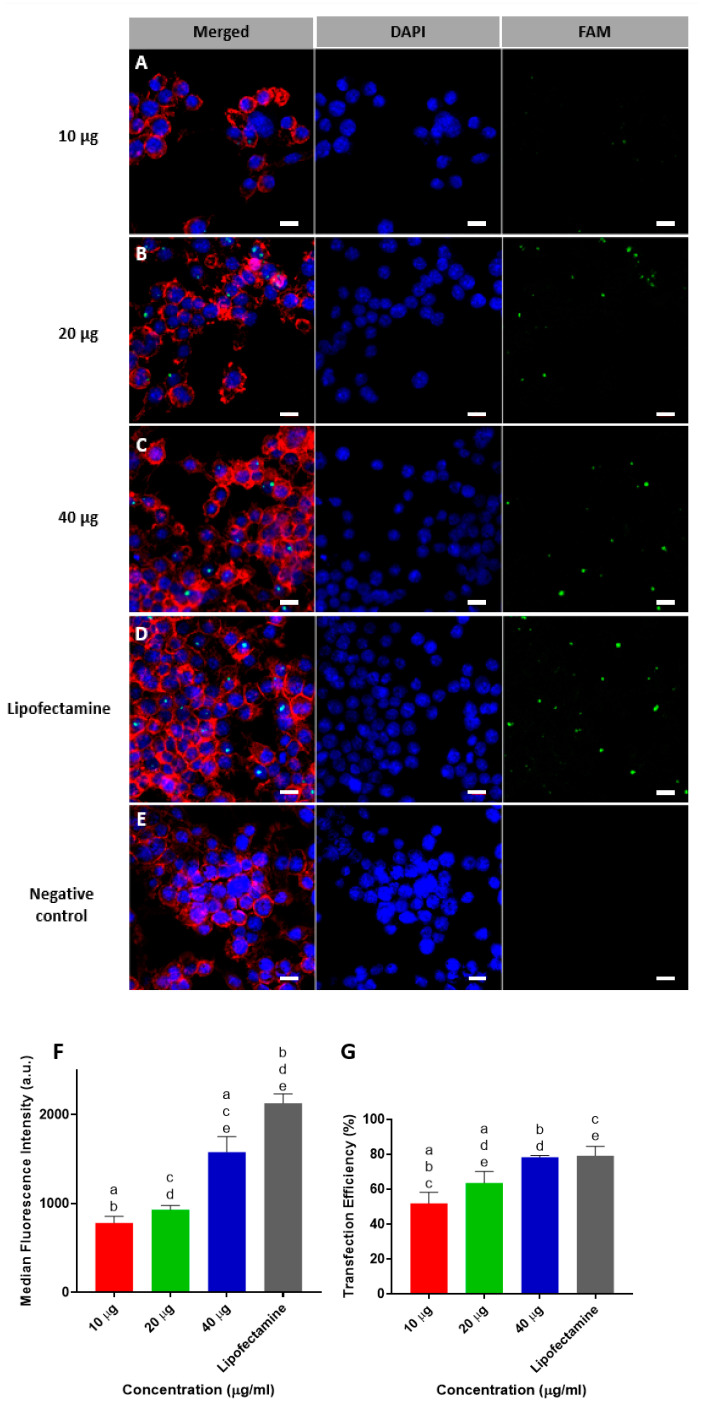
Transfection of RAW264.7 cells using FAM-labelled miRNA delivered using MSN-CC-PEI. Representative confocal microscopy images are shown for cells exposed to 10, 20, and 40 µg MSN-CC-PEI (**A**–**C**) versus Lipofectamine 3000 (**D**) as a positive control and untreated cells (**E**) as negative control. Cell nuclei were stained with DAPI (blue), and the cytoskeleton was stained with phalloidin (red). Panels (**A**–**C**) show green fluorescing RAW264.7 cells after successful transfection with FAM-labelled microRNA (green) using nanoparticles for 6 h (Scale bar = 10 µm). Data from flow cytometry analysis for mean fluorescence intensity and percentage transfection efficiency are shown in panels (**F**) and (**G**), respectively (*n* = 3). Results are shown as mean ± SD. Negative control used as calibrating base line and its fluorescence intensity and efficiency was equal to zero. Same letters indicate *p* < 0.05 via post hoc Tukey tests (*n* = 3).

Confocal microscope images of transfected RAW264.7 cells that had been incubated with 10, 20, or 40 µg of MSN-CC-PEI-FAM-miRNA are shown in Figure 3 from panels A to E. Following 6 h of incubation, green fluorescent dots were observed in the cytoplasm of macrophages. Lipofectamine was used as a gold standard. There was dose-dependent internalization of the nanocomplexes, with a high level of internalization at a concentration of 40 μg/mL, which was comparable to that of Lipofectamine. Moreover, the fluorescence intensity and the percentage transfection efficiency also were maximal for a concentration of 40 μg/mL (1580 a.u. ± 173 and 78.3% ± 0.7, respectively) with no significant difference compared to that of Lipofectamine (2130 a.u. ± 105 and 79.2% ± 3.7, respectively) (Figure 3 from panels F and G). Based on the cell viability results and our previous data for a balance between transfection efficiency and cell viability [19], MSN-CC-PEI were used at a concentration of 20 μg/mL for further experiments.

### 3.3. Modulatory Effects of MSN-CC-PEI-miRNA-26a on Macrophages

**Figure 4:**
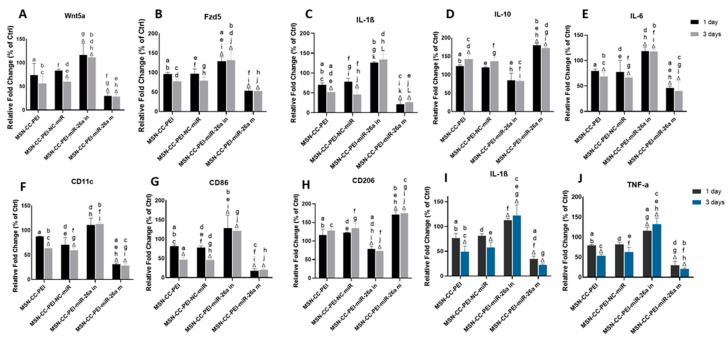
Immunomodulatory effects of MSN-CC-PEI-miRNA-26a on RAW264.7 cells, showing the gene expression profile (panels **A**–**H**) and protein expression (panels **I** and **J**) after treatment with MSN-CC-PEI, the MSN-CC-PEI-negative control (NC)-miRNA, MSN-CC-PEI-miRNA (miR)-26a inhibitory (in), or the MSN-CC-PEI-miRNA-26a mimic (m) for 1 day (black bars) and 3 days (grey/blue bars). Results are shown as mean ± SD. Δ indicates *p* < 0.05, which was compared with the control (Ctrl) group via post hoc Tukey tests (*n* = 3). The control group was composed of cells only, without nanoparticles or miRNA. Similar letters indicate *p* < 0.05 and significant differences between groups via post hoc Tukey tests (*n* = 3).

The inflammatory response of macrophages was determined via qRT-PCR (Figure 4 from panels A to H) and via an immunoassay (Figure 4, panels I and J). The expression of *Wnt5a* and *Fzd5* were downregulated by the MSN-CC-PEI-miRNA-26a mimic and were upregulated by the MSN-CC-PEI-miRNA-26a inhibitor (*p* = 0.001) (Figure 4, panels A and B). 

Subsequently, the macrophage expression of IL-1β and IL-6 was also downregulated by the miRNA-26a mimic and upregulated by the miRNA-26a inhibitor (*p* < 0.001) (Figure 4, panels C and D). MSN-CC that were devoid of cargo (i.e., without functional miRNA) also caused a downregulation in *Wnt5a*/Ca^2+^ pathway-related genes and in IL-1β and IL-6. The cytokine levels were significantly lower than those in control groups after 3 days for IL-1β and IL-6 in both the MSN-CC-PEI and MSN-CC-PEI-NC-miRNA groups (*p* = 0.03) (Figure 4, panels C and D). In contrast, the expression of IL-10 was upregulated in all groups, except the miRNA-26a inhibitory one (Figure 4 panel E). 

The expression levels of the two typical M1 macrophage markers CD11c and CD86 were significantly reduced by the miRNA-26a mimic (*p* = 0.001). Of interest, MSN-CC groups without functional miRNA also caused a distinct reduction of CD11c and CD86 levels after 3 days (*p* < 0.05) (Figure 4, panels F and G). On the other hand, the miRNA-26a mimic and inhibitor had an opposite function on the expression of CD206. The miRNA-26a mimic group showed a significant upregulation of CD206, whereas this marker was downregulated by the miRNA-26a inhibitor (*p* < 0.05) (Figure 4, panel H). Although MSN-CC-PEI also increased CD206 to a small extent, this was not statistically significant. 

Immunoassays for IL-1β and TNF-α showed a reduction of these pro-inflammatory cytokines due to the use of the miRNA-26a mimic (*p* = 0.001) (Figure 4, panels I and J). Moreover, MSN-CC-PEI without functional miRNA also decreased the levels of IL-1β and TNF-α. Both qRT-PCR and ELISA findings showed a time-dependent effect for MSN-CC-PEI on macrophages. However, there was no significant difference between 1 and 3 days within the other treatment groups.

### 3.4. Effects of the Modulated Immune Environment on the Osteogenic Differentiation of Preosteoblasts

**Figure 5:**
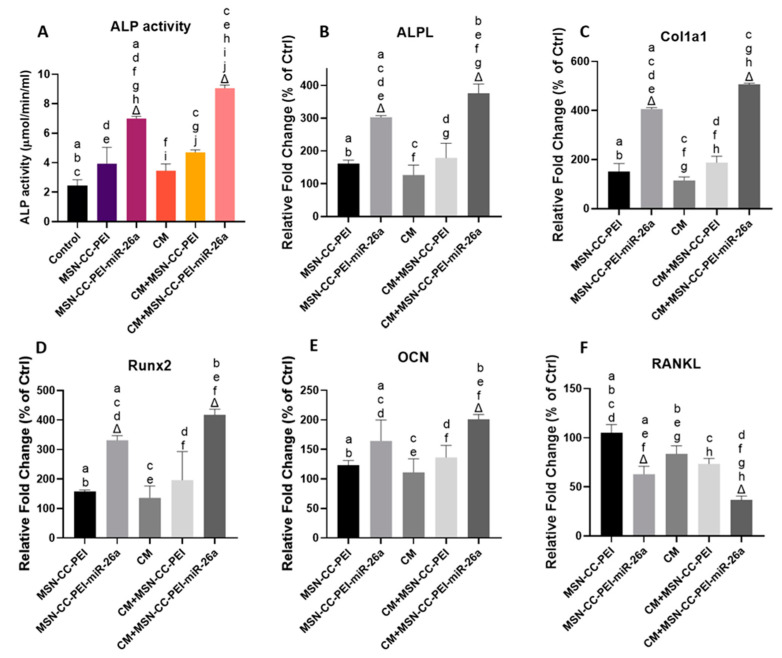
ALP activity (panel **A**) and gene expression (panels **B**–**F**) for MC3T3-E1 cells after treatment with MSN-CC-PEI or MSN-CC-PEI-miRNA (miR)-26a with or without conditioned medium (CM) for 14 days. Results are shown as mean ± SD. Δ indicates *p* < 0.05, which was compared with the control (Ctrl) group. The control group was cells not treated with nanoparticles or miRNA. Similar letters indicate *p* < 0.05 and significant differences between groups via post hoc Tukey tests (*n* = 3).

**Figure 6:**
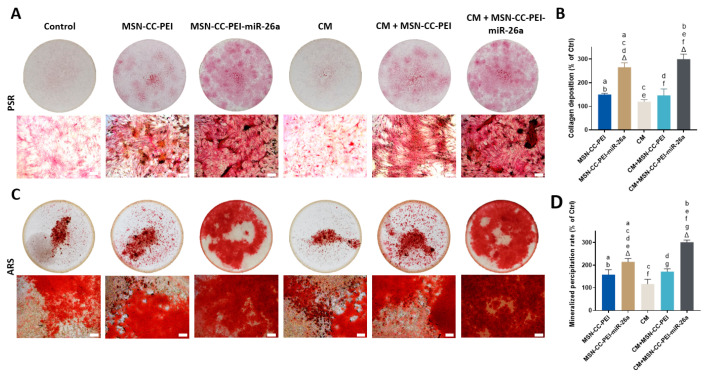
Collagen secretion and mineral deposition by MC3T3-E1 cells shown via staining with Picrosirius red (panels **A** and **B**) and Alizarin red (panels **C** and **D**). MC3T3-E1 cells were treated with MSN-CC-PEI or MSN-CC-PEI-miRNA-26a with or without conditioned medium (CM). Picrosirius red staining after 14 days is shown in panel A for each experimental group. Circles show images of the wells at a low magnification, while a high magnification view (×20) is shown beneath (scale bar = 100 µm). Panel **C** shows Alizarin red staining of mineralized nodules after 21 days. Picrosirius red staining and Alizarin red staining were quantified via spectrophotometry (panels **B** and **D**, respectively). Δ indicates *p* < 0.05, which was compared with the control (Ctrl) group. The control group was cells only without nanoparticles or miRNA. Similar letters indicate *p* < 0.05 and significant difference between groups via post hoc Tukey tests (*n* = 3).

To further investigate the ability of MSN-CC-PEI-miRNA-26a to exert osteoimmunomodulatory effects, we tracked alterations in ALP activity (Figure 5A), representative osteogenic markers (Figure 5, from panels B to F), extracellular matrix formation (Figure 6, panels A and B), and mineralization (Figure 6, panels C and D) in the presence of the conditioned medium from macrophages. The osteogenic behavior of MC3T3-E1 cells cultured with MSN-CC-PEI ± miRNA-26a in a standard medium or the corresponding macrophage conditioned medium [24] revealed the highest ALP, *ALPL*, Col1a1, and Runx2 activity levels in the MSN-CC-PEI-miRNA-26a + CM group (Figure 5, from panels A to D). 

Nevertheless, MSN-CC-PEI-miRNA-26a with CM demonstrated significantly enhanced ALP, *ALPL*, and Col1a1 activity levels compared with those of MSN-CC-PEI-miRNA-26a without CM (*p* < 0.05) (Figure 5, from panels A to C). However, the fold increases in the expression of *Runx2* and *OCN* genes in MC3T3-E1 cells cultured with MSN-CC-PEI-miRNA-26a in the corresponding CM did show a significant improvement in comparison with those of MSN-CC-PEI-miRNA-26a in standard medium (Figure 5 panels D and F). In MSN-CC-PEI + standard medium, the ALP, *ALPL*, *Col1a1*, *Runx2*, and *OCN* activity levels showed only slight enhancement compared with those of the control group, and the difference was not statistically significant (*p* > 0.05). Consistent with the results of the immunoassay regarding the expression of TNF-α from macrophages, the RANKL levels in both MSN-CC-PEI-miRNA-26a groups decreased substantially, with the lowest level being in the MSN-CC-PEI-miRNA-26a + CM group (Figure 5F).

The formation of collagen matrix and mineralization nodules is considered to be key evidence of in vitro osteogenesis. Picrosirius red and Alizarin red stains were used to visualize the collagen matrix and mineralization after 14 and 21 days, respectively (Figure 6, from panels A to D). A similar trend was found for both stains, with the highest amount of collagen deposition and mineralization being seen in the MSN-CC-PEI-miRNA-26a + CM group, which was consistent with the gene expression profile for this same group. Quantitative assessments revealed that groups with MSN-CC-PEI-miRNA-26a (±CM) had significantly elevated collagen deposition and mineralized nodule formation compared to those of the control (*p* < 0.05) (Figure 6, panels B and D). Moreover, using macrophage-conditioned medium from cells exposed to MSN-CC-PEI-miRNA-26a increased the rate of mineral deposition compared to that of the standard culture medium (Figure 6D) (*p* = 0.016).

## 4. Discussion

This study shows the powerful actions of MSN-CC-PEI as a nanoparticle vector for carrying miRNA-26a. This particular microRNA has been reported to enhance osteogenesis via boosting the proliferation and differentiation of mesenchymal stem cells acting via the Wnt pathway [19], as well as exerting immunomodulatory actions, such as in cancer immunotherapy [25,26]. In this study, we further demonstrated that these nanoparticles could efficiently transfect both macrophages and preosteoblasts and that miRNA-26a has immunomodulatory effects in osteogenesis, including suppressing the acute inflammatory response from macrophages and promoting the osteogenic differentiation of preosteoblasts. 

To better understand the influence of macrophages on osteogenesis, the present study focused on in vitro interactions between preosteoblasts and macrophages after transfection with MSN-CC-miRNA-26a. The findings support previous work demonstrating the role of miRNA-26a in bone regeneration [19], and we expand on it by showing, for the first time, the effect of miRNA-26a on osteoimmunomodulation. To expand on this work, additional investigations of safety issues are needed prior to in vivo investigations, even though we found excellent levels of macrophage viability within the correct therapeutic dose range (Figure 2, panel D). As was expected, PEI coating increased the cytotoxicity of MSNs [27] (Figure 1, panels A to C); however, MSN-CC-PEI did not cause issues with the viability of RAW 264.7 macrophage cells when the concentration was kept below 40 µg/mL. The biocompatibility of nanoparticles is greatly affected by their morphology, size, surface charge, and surface modifications (reviewed in [20]). MSNs with a monodispersed mesoporous structure have a positive effect on the adhesion and proliferation of cells, including mesenchymal stem cells, and they have a high level of biocompatibility over. These features make them attractive as a non-viral vector [19,20].

### 4.1. Immunomodulatory Effects of miRNA-26a-MSN-CC-PEI on Macrophages

Due to the multiple impacts of macrophages on bone healing processes, in this study, we explored how miRNA-26a influenced RAW 246.7 murine-derived macrophages, as these are a well-known model used in cell culture studies [28]. The first part of assessing osteoimmunomodulation was to examine the response of macrophages to transfection with miRNA-26a. The assessment of *Wnt5a*/Ca^2+^ pathway-related genes (*Wnt5a* and *Fzd5*) showed the significant downregulation via the miRNA-26a mimic, and this finding was consistent with *Wnt5a* and *Fzd5* upregulation by the miRNA-26a inhibitor. The *Wnt5a*/Ca^2+^ pathway activates NF-κB signaling, which leads to the secretion of pro-inflammatory cytokines (e.g., IL-1β and IL-6) and drives inflammation [29,30]. The present findings show that miRNA-26a strongly suppresses the production of pro-inflammatory cytokines, which is consistent with the suppression of *Wnt5a* and *Fzd5*, which participate in the *Wnt5a*/Ca^2+^ pathway. Previous studies have demonstrated similar alterations caused by miRNA-26 [31,32,33]. For instance, miRNA-26 inhibits the immune response in *Mycobacterium tuberculosis* infection via the suppression of NF-κB signaling [32]. The overexpression of miRNA-26a causes lowered production of IL-6 and TNF-α by microglial cells [31]. In addition, miRNA-26a can influence the expression of Toll-like receptors in inflammatory states, as shown in a rat arthritis model for Toll-like receptor 3 protein expression [33]. 

**Figure 7:**
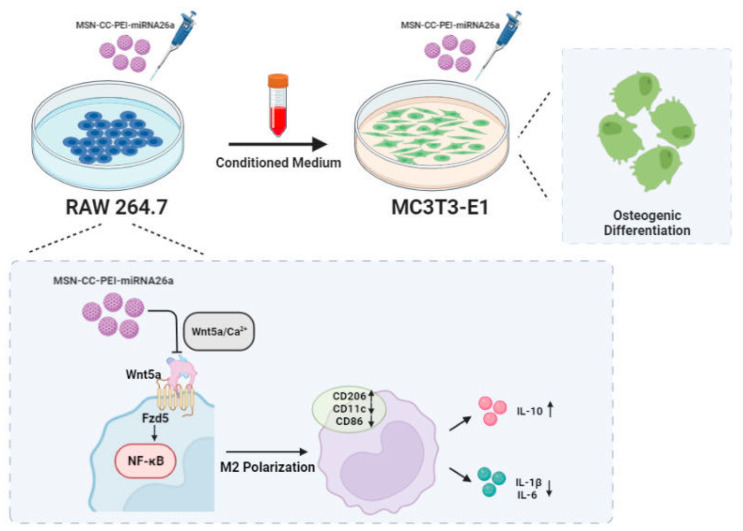
MSN-CC-PEI-miRNA26a promoted osteogenic differentiation of MC3T3-E1 via affecting the osteoimmune environment via modulating the responses of RAW 264.7 cells.

The present study showed the potent effects of miRNA26 delivered by MSC-CC-PEI on macrophages (Figure 7). Based on their surface markers and functional properties, macrophages are broadly subtyped into M1 and M2 phenotypes [34]. Both M1 and M2 macrophages influence bone healing. During early events, M1 macrophages, which express CD11c and CD86, secrete pro-inflammatory cytokines that stimulate osteoclasts [9]. M1 macrophages also induce preosteoblasts to differentiate into fibroblasts [35], and M1 macrophages secrete TNF-α, which inhibits mineralization and osteogenic differentiation [36,37]. On the other hand, M2 macrophages are involved in the late stages of tissue healing [38]. Previous work has shown that several miRNAs are involved in macrophage polarization [15], with miRNA-125a, miRNA-511-5p, and miRNA-92a promoting M2 polarization [15]. The present findings indicate that miRNA-26a drives RAW 264.7 macrophages towards M2, with upregulated CD206 and downregulated CD11c and CD86 and the reduced expression of pro-inflammatory cytokines at the gene and protein levels. As well, it causes greatly enhanced levels of IL-10, which is an anti-inflammatory cytokine released by M2 macrophages. 

The present results show the importance of the concentration of nanoparticles. Previous studies have given conflicting results regarding the responses induced by MSNs (reviewed in [39]), with some describing acute inflammatory responses due to oxidative stress-related changes [40,41], and others describing reduced expression of pro-inflammatory genes [42,43,44]. All these actions are dose-dependent. This is not surprising, as high concentrations of MSN can activate the NF-κB signaling due to oxidative stress and cytotoxicity [45]. However, at low concentrations, the degradation of MSNs will gradually release Si ions, which suppress inflammation [43]. In our previous research, we did show that MSN biodegradation will release Si ions for up to 4 days [46]. The activation of the canonical WNT5A/Ca^2+^ pathway by silicon suggests that the released Si ions from the degradation of MSNs play a crucial role in inhibiting inflammation [39]. Furthermore, MSNs have the potential to enhance the secretion and release of IL-4 and IL-10 from type 1 and type 2 T helper lymphocytes, respectively [39]. Both of these interleukins possess anti-inflammatory properties. Consequently, although MSNs may initially activate the NF-κB pathway, this effect is transient and lasts only for a few hours. Once the degradation of particles begins, anti-inflammatory effects become evident [39]. Consistent with this explanation, the present study showed, at the gene and protein levels, the downregulation of TNF-α, IL-1β and IL-6, especially after 3 days, as well as the upregulation of IL-10. This was linked to altered expression of CD11c, CD86, and CD206, as the cells polarized toward the M2 phenotype, which is, once again, in line with previous results [43,47]. The underlying mechanism could be related to the Wnt5a/Ca^2+^ pathway. Autophagy regulates macrophage polarization [48] and can participate in the effects of MSN on macrophage polarization [43]. 

### 4.2. Immunotherapeutic Effects of MSN-CC-PEI-miRNA-26a on Preosteoblasts

As mentioned earlier, miRNAs play a central role in the immune system [49], and several miRNAs have been identified as regulators of key pathways, including TLR, NF-κB, and TGF-β [50,51]. For bone healing, a pro-inflammatory reaction following an injury is an essential trigger or initiator of the healing process [52]. However, a pronounced or prolonged pro-inflammatory reaction impairs the healing process [53,54]. The current findings show that the microenvironment created by MSN-CC-PEI-miRNA-26a, as shown within the indirect co-culture model, has a positive impact on osteogenesis.

Transfection using MSN-CC-PEI-miRNA-26a lowered the expression level of pro-inflammatory cytokines by macrophages and promoted a shift in these cells away from acute inflammation. The crosstalk between these macrophages and preosteoblasts, which share several signaling pathways, explains how osteogenic differentiation is promoted [55]. The osteoimmune environment in the present study consisted of three components: 1. The immunomodulatory effect of the MSN-CC-PEI-miRNA-26a mimic on macrophages and the resulting released cytokines; 2. The direct effect of the miRNA-26a mimic on MC3T3-E1 cells; 3. The effects of MSN-CC-PEI and its degradation by-products on both cell types. 

A conditioned medium derived from LPS-activated M1 macrophages will contain inflammatory cytokines, such as IL-1β, IL-6, and TNF-α, and these will trigger preosteoblasts to differentiate into fibroblasts [24], while suppressing the expression of osteogenic markers and mineralization [37,45]. This is how local infections cause failures in sites where bone tissue engineering techniques have been used [35]. Conversely, based on the immunomodulatory effects of the MSN-CC-PEI-miRNA-26a mimic seen in this study, inhibiting excessive inflammation can support enhanced in vitro osteogenesis. 

We assessed the expression of TNF-α from macrophages and RANKL from MC3T3-E1 cells. As a ligand for receptor activators of nuclear factor kappa-Β (NFkB), RANKL is secreted by osteoblasts and mesenchymal stem cells to initiate osteoclastic activity. RANKL is also a member of the TNF superfamily [56]. In the present study, miRNA-26a suppressed the *Wnt5a*/Ca^2+^ pathway in macrophages. The resulting suppression of NFkB explains the reduction of the gene and protein expression levels of RANKL and TNF-α in both cell types. We conclude that through a combined effect on macrophages and osteoblasts, miRNA-26a inhibits osteoclast formation via decreasing the amount of RANKL.

miRNA-26a has a direct effect on mesenchymal stem cells [56,57,58,59] and promotes their osteogenic differentiation [60,61,62,63]. In a previous study, we showed that miRNA-26a promotes osteogenesis for rat bone marrow mesenchymal stem cells (BMSC), which was consistent with other reports [61,62,63]. The explanations for this include its impact on intrinsic signal networks in these BMSCs, which may respond in different ways than other stem cells do [61]. Glycogen synthase kinase 3 (GSK3) is a serine/threonine kinase involved in regulating glycogen deposition, while GSK3β is a key negative regulator of the canonical Wnt/β-catenin and PI3K/Akt signaling pathways [64]. Both pathways are involved in promoting osteogenesis [65,66]. In the presence of Wnt ligands, GSK3β is inactivated, and this permits β-catenin to pass into the nucleus, where it regulates further gene expression along the Wnt/β-catenin pathway [67]. This explains the higher levels of osteogenic genes expressed via the induction of MSN-CC-PEI-miRNA-26a.

One must also point out that the nanovector itself (the MSN-CC-PEI delivery component) has significant impacts on macrophages, and this is in line with previous reports [43,68]. The key aspects include the enhanced expression of ALP and osteogenic markers, as well as in *ALPL* and *Col1a1* genes. When one is considering the osteoimmune environment, attention must also be paid to the effects of silicon ions released from degrading MSN, which could be present in the culture medium. This may explain why there was an elevated expression of ALP in the MSN-only group [69,70] and why adding CM to MSN-CC-PEI without functional miRNA caused the enhanced expression of all osteogenic genes. These aspects need further exploration using animal models.

## 5. Conclusions

The results of this study are promising in terms of how a nanovector (MSN-CC-PEI) could potentially be used for enhancing bone regeneration via manipulating the microenvironment through the nanoparticle itself, as well as through its cargo of miRNA-26a. The latter way creates a highly osteoinductive environment via altering the responses of macrophages, driving these away from an inflammatory phenotype, as well as the behavior of preosteoblasts, driving these towards mineral deposition. The nanovector used had low toxicity and efficient transfection properties. Together, these findings show how the influence of a nanomaterial can be directed towards a beneficial overall impact on a complex biological process, in this case, osteogenesis, and ultimately, bone regeneration. Although studies have already highlighted the potential adverse effects of MSNs, including acute inflammatory responses induced via oxidative stress-related changes, future investigations focusing on evaluating the genotoxicity and oxidative stress induced by low concentrations of MSN-CC-PEI nanoparticles would provide valuable insights.

## Figures and Tables

**Table 1:** Primer sequences used in this study for qRT-PCR assessment.

Gene Bank	Gene	Forward Reverse
NM_001256105.1	*Wnt5a* *Wnt5a*	5′-CAACTGGCAGGACTTTCTCAA-3′ 5′-CCTGATACAAGTGGCAGAGTTTC-3′
NM_001042659.1	*Fzd5*	5′-AAGTCCATTACGGCGCTG-3′ 5′-AGCCTCGTAGTGAGTTCAGG-3′
NM_008361.4	*IL-1β*	5′-TGGAGAGTGTGGATCCCAAG-3′ 5′-GGTGCTGATGTACCAGTTGG-3′
NM_001314054.1	*IL-6*	5′-ATAGTCCTTCCTACCCCAATTTCC-3′ 5′-GATGAATTGGATGGTCTTGGTCC-3′
NM_010548	*IL-10*	5′-AAGTCCATTACGGCGCTG-3′ 5′-AGCCTCGTAGTGAGTTCAGG-3′
NM_001363984.1	*CD11c*	5′-ACTTCACGGCCTCTCTTCC-3′ 5′-CACCAGGGTCTTCAAGTCTG-3′
NM_019388.3	*CD86*	5′-CTGCTCATCATTGTATGTCAC-3′ 5′-ACTGCCTTCACTCTGCATTTG-3′
NM_008625	*CD206*	5′-GTTCACCTGGAGTGATGGTTCTC-3′ 5′-AGGACATGCCAGGGTCACCTTT-3′
NM_053470.1	*RUNX2*	5′-GAGCACAAACATGGCTGAGA-3′ 5′-TGGAGATGTTGCTCTGTTCG-3′
NM_013059.1	*ALPL*	5′-GCACAACATCAAGGACATCG-3′ 5′-TCAGTTCTGTTCTTGGGGTACAT-3′
NM_053304.1	*Col1α1*	5′-GCAACAGTCGCTTCACCTACA-3′ 5′-CAATGTCCAAGGGAGCCACAT-3′
M25490.1	*OCN*	5′-TCTTTCTCCTTTGCCTGGC-3′ 5′-CACCGTCCTCAAATTCTCCC-3′
NM_003701.4	*RANKL*	5′-TGTACTTTCGAGCGCAGATG-3′ 5′-AGGCTTGTTTCATCCTCCTG-3′
NM_017008.4	GAPDH *	5′-TGTGTCCGTCGTGGATCTGA-3′ 5′-TTGCTGTTGAAGTCGCAGGAG-3′

* Housekeeping gene.

## Data Availability

We already included all results in the manuscript and there is no additional data to share.
